# Sex-Related Differences in Voluntary Alcohol Intake and mRNA Coding for Synucleins in the Brain of Adult Rats Prenatally Exposed to Alcohol

**DOI:** 10.3390/biomedicines10092163

**Published:** 2022-09-02

**Authors:** Viktor S. Kokhan, Kirill Chaprov, Natalia N. Ninkina, Petr K. Anokhin, Ekaterina P. Pakhlova, Natalia Y. Sarycheva, Inna Y. Shamakina

**Affiliations:** 1 V.P. Serbsky Federal Medical Research Centre for Psychiatry and Narcology, 119034 Moscow, Russia; 2 Institute of Physiologically Active Compounds RAS, 142432 Chernogolovka, Russia; 3 Belgorod State National Research University, 308015 Belgorod, Russia; 4 Lomonosov Moscow State University, 119991 Moscow, Russia

**Keywords:** prenatal alcohol exposure, synucleins, alcohol consumption, free-choice, hippocampus, midbrain, mRNA expression, α-synuclein, rats

## Abstract

Maternal alcohol consumption is one of the strong predictive factors of alcohol use and consequent abuse; however, investigations of sex differences in response to prenatal alcohol exposure (PAE) are limited. Here we compared the effects of PAE throughout gestation on alcohol preference, state anxiety and mRNA expression of presynaptic proteins α-, β- and γ-synucleins in the brain of adult (PND60) male and female Wistar rats. Total RNA was isolated from the hippocampus, midbrain and hypothalamus and mRNA levels were assessed with quantitative RT-PCR. Compared with naïve males, naïve female rats consumed more alcohol in “free choice” paradigm (10% ethanol vs. water). At the same time, PAE produced significant increase in alcohol consumption and preference in males but not in females compared to male and female naïve groups, correspondingly. We found significantly lower α-synuclein mRNA levels in the hippocampus and midbrain of females compared to males and significant decrease in α-synuclein mRNA in these brain areas in PAE males, but not in females compared to the same sex controls. These findings indicate that the impact of PAE on transcriptional regulation of synucleins may be sex-dependent, and in males’ disruption in α-synuclein mRNA expression may contribute to increased vulnerability to alcohol-associated behavior.

## 1. Introduction

Alcohol use disorder (AUD) is a severe and etiologically complex disease associated with high morbidity and mortality rates. Although the development of alcohol dependence has a strong genetic component [1], there are a wide variety of environmental factors that impact the vulnerability to AUD through epigenetic modifications [2].

Maternal alcohol drinking leading to prenatal alcohol exposure (PAE) is one of the strong predictive factors of AUD [3]. Neurobehavioral disorder associated with prenatal alcohol exposure can occur in children following even low to moderate levels of maternal ethanol consumption during pregnancy and touch on the basic components of cognition—learning, memory, executive functions, affective state anxiety and depression, and addictive behavior later in life [4,5]. To date the epigenetic mechanisms underlying high risk of drug abuse which arise from maternal consumption of alcohol are unclear. It is well known that alterations in presynaptic regulation of neurotransmission are among key mechanisms involved in the long-lasting ethanol effects, excessive drinking and ethanol abuse [6]. Central component of the presynaptic neurotransmitter release machinery is soluble NSF attachment protein receptor complex, which in turn is under regulatory control of synucleins—small presynaptic proteins that are expressed from three genes (α-, β- and γ-synuclein) and may modify neurotransmitter release [7]. α-Synuclein has been previously studied as a potential player in alcohol addiction due to its role in dopamine (DA) neurotransmission [8]. Genome-wide association studies have found α-synuclein to be one of the candidate gene for alcoholism, and single-nucleotide polymorphisms in intron 4 of the α-synuclein gene (*Snca*) have been linked to alcohol craving [9]. It has been found that variability in *Snca* which affects its expression may contribute to the high risk of alcohol abuse [10]. It was shown that elevated α-synuclein mRNA levels are associated with craving in patients with alcoholism [11,12]; transgenic mice expressing human mutant A30P α-synuclein were characterized by higher motivation for ethanol and attenuated context- and cue-induced reinstatement of alcohol-seeking behavior [13]. At the same time, alcohol-dependent subjects had higher frequencies of the shortest 267 bp allele of the α-synuclein-repeat 1 marker, associated with decreased expression of α-synuclein in the autopsy samples of human prefrontal cortex [10]. These and other data have inspired the α-synuclein deficit hypothesis: low basal levels of α-synuclein in some brain regions may predispose to enhanced DA activity in response to alcohol and as a result alcohol cravings and excessive alcohol consumption, leading in turn to increases in α-synuclein [9]. This hypothesis is in accordance with the fact, that α-synuclein expression is downregulated in the frontal cortex and caudate–putamen of inbred alcohol preferring rats prior to ethanol exposure [14].

β-Synuclein—a pre-synaptic protein that co-localizes with α-synuclein, can act as α-synuclein inhibitor [15] and suggested to have neuroprotective properties [15]. Mammalian γ-synuclein is mainly expressed in the peripheral nervous system (primary sensory, sympathetic and motor neurons), but also detected in the brain [16]. The normal cellular function of γ-synuclein still remains unknown. Interestingly, γ-synuclein knockout mice were hypoactive in a novel environment [17], whereas α- and γ-synuclein double-null knockout mice were hyperactive due to the hyperdopaminergic phenotype detected by two-fold increase in the extracellular concentration of dopamine after electrical stimulation of striatum [18].

Considering that dopaminergic imbalance in the brain can be one of the determinants of high alcohol motivation [19,20], and synucleins may be critically involved in regulation of dopaminergic neurons [8] here we studied alterations in synucleins mRNA level in specific brain areas of adult male and female rats affected by prenatal alcohol exposure. It was of particular importance to focus on the sex-dependent alterations in gene expression which might provide more insight into the problem of sex differences in response to alcohol intake both in humans [21,22] and animal models of alcoholism [23,24].

## 2. Materials and Methods

### 2.1. Animals

**Figure 1:**
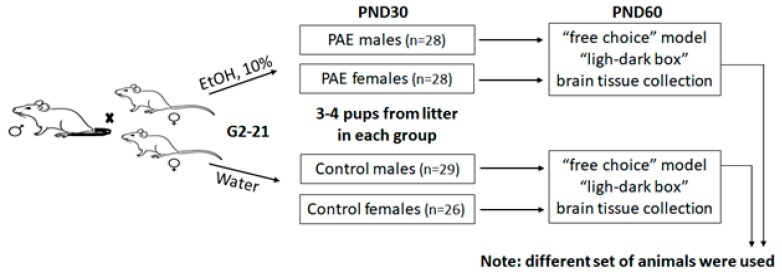
Scheme of the experimental design. EtOH—ethanol. PAE—prenatal alcohol exposure. PND—postnatal day.

Adult (postnatal day 60; PND60) male and female Wistar rats were supplied by Stolbovaya Animal Farm (Moscow region, Russia) and group-housed 5 per a cage in a 12/12-h light/dark cycle, 19–22 °C and 55% humidity with free access to water and standard lab chow. Following a one-week acclimation period in the animal facility two female rats were matched with one male for 72 h, thus the number of rats in one cage was 3 (2♀ + 1♂). In the current experiment conception occurred 1–2 days following male-female pairing. After confirmation of pregnancy by the presence of a vaginal plug male partners were removed and females were singly housed until giving birth. In this case, 20 female rats were distributed into two groups equally (10 animals/group): ethanol-exposed (received 10% (*v*/*v*) ethanol as the only fluid, E2-birth, *n* = 10) and intact (received water as the only fluid all the time, *n* = 10). A gestational period “E2-birth” is analogous to the first two trimesters of human gestation [25]. The human third trimester equivalent occurs in rodents following birth (PND 1–10) [26,27], thus, in accordance with the goal of the study (prenatal alcohol exposure study) during this period, the offspring/dams did not receive ethanol. Offspring were weaned at PND21 and housed 5 rats of the same sex in one cage. At postnatal day 60 (PND 60) male and female offspring were divided into three groups and used for: (1) test for anxiety (“light-dark box”), (2) test for alcohol preference, (3) analysis of mRNA expression. For each test we used equal number of rats per sex per litter from both PAE and control groups. Each experimental group consisted of no more than 3 littermates. A scheme of the experimental design is shown in Figure 1.

### 2.2. Two-Bottle “10% Alcohol vs. Water” Choice Drinking Paradigm (Voluntary Alcohol Consumption)

At PND 60 rats were housed individually in standard cages (43.5 × 28 × 16 cm) and presented with 2 bottles containing diluted ethanol (10% *v*/*v*) and the other containing water, providing a 24-h continuous free-choice alcohol access. Alcohol and water intake were carefully measured by weighing the bottles every 24 h at 8 a.m. (0.1 g accuracy). The calculated parameters were daily intake levels of water, 10% Alcohol and total fluid. Alcohol preference was estimated as a ratio of 10% Alcohol vs. total daily fluid consumed (10% Alcohol/Alcohol + water). There were four groups: control male offspring from intact dams (C-m, *n* = 11), PAE male offspring (PA-m, *n* = 10), control female offspring from intact dams (C-f, *n* = 8), PAE female offspring (PA-f, *n* = 10).

### 2.3. Light-Dark Box

Anxiety-like behavior was measured via light-dark box test (TSE, Berlin, Germany). Each rat was initially placed in the middle compartment (length× width × height, 13 × 21 × 35 cm) and monitored for 15 min in the box with free choice to move between and within brightly illuminated (left) and dark (right) compartments (both 21 × 21 × 35 cm). The following parameters were detected for each of the box compartments: number of entrances (full-body transitions between chambers), total distance, and the total spent time. There were four groups: control male offspring from intact dams (C-m, *n* = 9), PAE male offspring (PA-m, *n* = 9), control female offspring from intact dams (C-f, *n* = 9), PAE female offspring (PA-f, *n* = 9).

### 2.4. Tissue Collection

The animals from control male offspring from intact dams (C-m, *n* = 9), PAE male offspring (PA-m, *n* = 9), control female offspring from intact dams (C-f, *n* = 9) and PAE female offspring (PA-f, *n* = 9) were euthanized by decapitation and the following brain morphological structures were isolated on ice: hippocampus (HPC), hypothalamus (HPY) and midbrain (MID). The structures were immediately frozen in liquid nitrogen.

### 2.5. RNA Extraction, cDNA Synthesis and Quantitative RT-PCR

Total RNA was extracted using Extract RNA (Evrogen, Moscow, Russia) according to the standard phenol-chloroform protocol. DNAse (Thermo Scientific, Mundelein, IL, USA) was used for RNA purification and 1μg of total RNA was used for cDNA synthesis. Reverse transcription was performed using the MMLV-RT kit (Evrogen, Moscow, Russia) with random hexamer primers. Gene expression levels were analysed via real-time polymerase chain reaction (qRT-PCR) using qPCRmix-HS SYBR (Evrogen). The reaction was carried out in a BioRad CFX96 thermal cycler (Bio-Rad Laboratories Inc., California, USA). Relative gene expression values were obtained by using 2^−ΔΔCT^ method with β-actin as a reference gene. Primer sequences are shown in Table 1.

### 2.6. Data Analysis

The data were represented as the mean ± standard deviation (SD) and analyzed with the Statistica 12 software (StatSoft Inc., Tulsa, OK, USA). The datasets were tested for normality with the Shapiro-Wilk test (W-test) and the parametric analysis was applied if *p* > 0.05. The data obtained in the “alcohol intake” test were analyzed with repeated measures ANOVA. For the other data sets the two-way ANOVA was applied. The post-hoc Duncan’s multiple range test was used when appropriate.

## 3. Results

### 3.1. Observation of Pregnant Dam and Offspring

The mean maternal ethanol consumption was recorded daily throughout pregnancy and was 2.6 ± 0.72 g/kg per 24 h. This moderate ethanol exposure did not affect maternal weight gain and pregnancy outcomes—litter size, number of pups born, postnatal mortality or offspring birth weight compared to control group (data not shown).

### 3.2. Voluntary Alcohol Consumption

Alcohol drinking groups had access to 10% ethanol and water in two-bottle-choice paradigm for 7 days.

**Figure 2:**
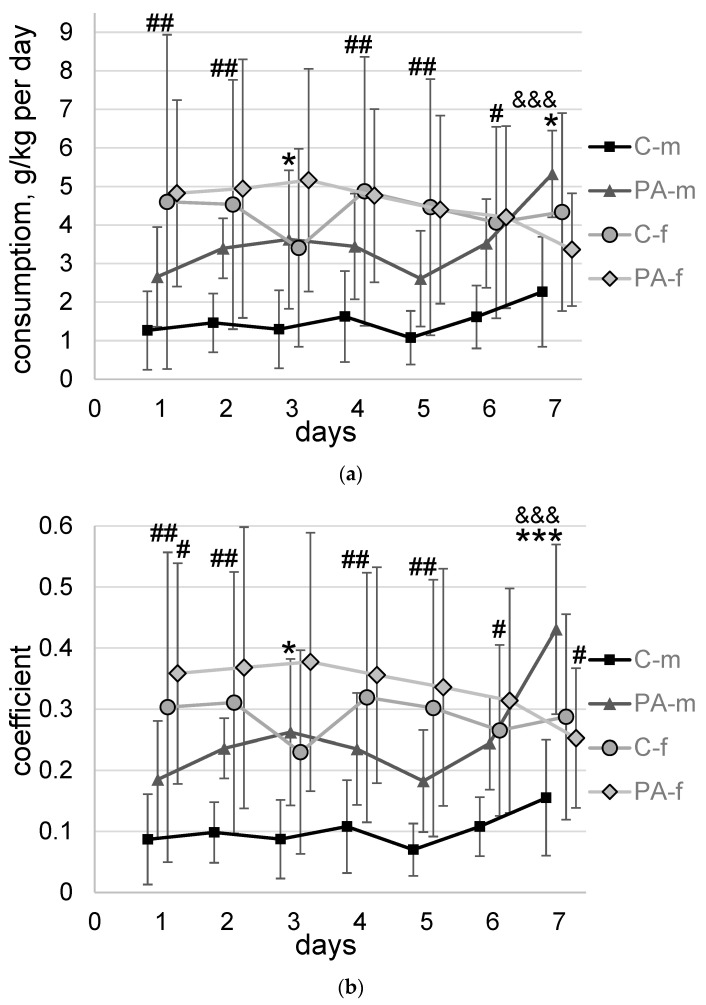
Two-bottle-choice paradigm. Data present as mean ± SD. (**a**)—alcohol consumption, g/kg of absolute ethanol per day; (**b**)—alcohol preference, coefficient: the ratio of alcohol consumed volume to total volume of alcohol and water consumed. C-m—male naïve rats, *n* = 11; PA-m—male PAE rats, *n* = 10; C-f—female naïve rats, *n* = 8; PA-f—female PAE rats, *n* = 10. Rats were provided with 24 h/day access to two-bottle choice drinking (one bottle contained 10% alcohol, the other contained water) for 7 days. Asterisk (*) indicates a significant difference between naïve and PAE rats of the same sex: *—*p* < 0.05, ***—*p* < 0.001; post hoc Duncan’s test. Hash (#) indicates a significant difference between groups of the same name of different sex: #—*p* < 0.05; ##—*p* < 0.01; post hoc Duncan’s test. Ampersand (&) indicates a significant difference between Day 1 and Day 7 inside a group: &&&—*p* < 0.001; post hoc Duncan’s test.

Statistically significant impact of PAE (F_1,35_ = 4.5, *p* = 0.04), sex (F_1,35_ = 13.7, *p* = 0.007), and interaction of alcohol consumption dynamics and sex (F_6,210_ = 3.8, *p* = 0.001) on alcohol consumption were found (Figure 2a). Estimation of daily ethanol intake indicated that C-f group of rats demonstrated higher alcohol consumption at Days 1, 2, 4, 5, and 6 (264%, *p* = 0.004; 209%, *p* = 0.009; 200%, *p* = 0.005; 314%, *p* = 0.004; 152%, *p* = 0.03, respectively) compared to C-m rats. The differences between the PA-f and PA-m groups were not found. At the same time, there was apparent difference in ethanol consumption between PAE and control (naïve) males, but not between PAE and naïve females. PA-m rats demonstrated significantly higher alcohol consumption at Day 3 and Day 7 of the experiment (180%, *p* = 0.04 and 135%, *p* = 0.009, respectively) compared to C-m rats. Among all experimental groups only PA-m rats showed a 101% (*p* = 0.0005) increase in alcohol consumption from Day 1 to Day 7 of the experiment.

Effects of PAE (F_1,35_ = 8, *p* = 0.008), sex (F_1,35_ = 14.5, *p* = 0.006), and interaction of alcohol preference dynamics and sex (F_6,210_ = 5.7, *p* = 0.00002), alcohol consumption dynamics, PAE and sex (F_6,210_ = 2.2, *p* = 0.04) on alcohol preference were found (Figure 2b). Estimation of daily drinking activity indicated that female rats in both PAE (only at the beginning of testing) and C groups prefer ethanol to water to a greater extent than males. C-f group of rats demonstrated higher alcohol preference at Days 1, 2, 4, 5, and 6 (249%, *p* = 0.007; 215%, *p* = 0.008; 196%, *p* = 0.008; 331%, *p* = 0.003; 146%, *p* = 0.046, respectively) compared to C-m rats. The differences between the PA-f and PA-m groups were somewhat less pronounced: PA-f group of rats demonstrated higher alcohol preference at Day 1—on 94% (*p* = 0.03), but lower at Day 7—(41%, *p* = 0.025) compared to PA-m rats. At the same time, there was apparent difference in the percentage preference for ethanol between PAE and naïve males, but not between PAE and naïve females. PA-m rats demonstrated significantly higher alcohol preference at Day 3 and Day 7 of the experiment (200%, *p* = 0.02 and 178%, *p* = 0.0005, respectively) compared to C-m rats. Along with the detected increase in alcohol consumption, PA-m rats showed a 133% (*p* = 3 × 10^−6^) increase in alcohol preference from Day 1 to Day 7 of the experiment, whereas for other groups, no statistically significant change in the dynamics of alcohol preference was found.

### 3.3. The “Light-Dark Box” Test

The following statistically significant differences were found in the “light-dark box” test: number of entries into the dark compartment (F_1,32_ = 6.1, *p* = 0.02; PAE and sex factor interaction), distance in the dark compartment (F_1,32_ = 4.2, *p* = 0.049; PAE and sex factor interaction), number of entries into the middle compartment (F_1,32_ = 8.2, *p* = 0.007; PAE and sex factor interaction). An effect of PAE (F_1,32_ = 5.8, *p* = 0.02) and PAE and sex factor interaction (F_1,32_ = 6.6, *p* = 0.015) was found, when total distance in all compartments was analyzed.

**Figure 3:**
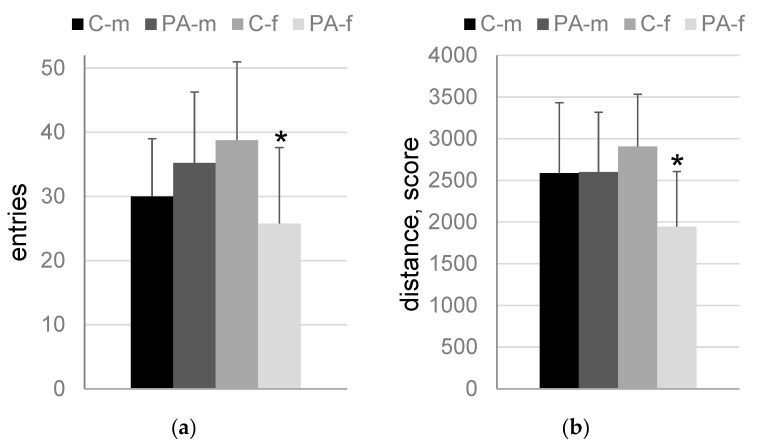

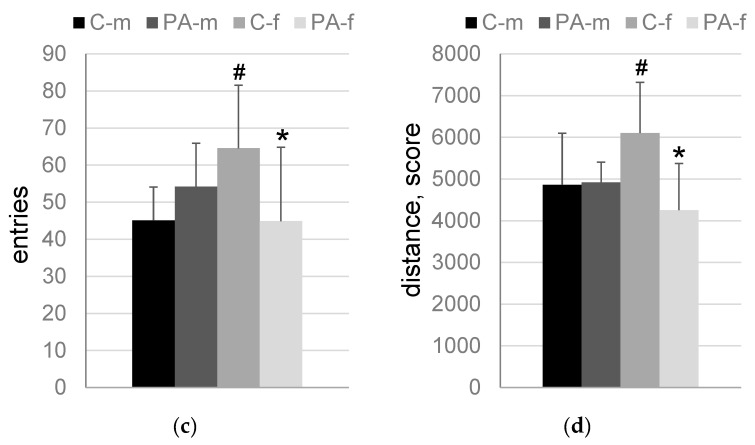
Light-dark box. Data present as mean + SD. (**a**)—entries into the dark compartment; (**b**)—distance in the dark compartment, scores; (**c**)—entries into the middle compartment; (**d**)—total distance travelled in all compartments, scores. C-m—male naïve rats, *n* = 9; PA-m—male PAE rats, *n* = 9; C-f—female naïve rats, *n* = 9; PA-f—female PAE rats, *n* = 9. Asterisk (*) indicates a significant difference between naïve and PAE rats of the same sex: *—*p* < 0.05; post hoc Duncan’s test. Hash (#) indicates a significant difference between groups of the same name of different sex: #—*p* < 0.05; post hoc Duncan’s test.

There were no significant differences between PA-m and C-m rats. At the same time, PA-f rats characterized by lower number of entries (66.5%, *p* = 0.03, Figure 3a) and distance (67%, *p* = 0.01, Figure 3b) in the dark compartment, number of entries into the middle compartment (70%, *p* = 0.015, Figure 3c) and total distance in all compartments (70%, *p* = 0.002, Figure 3d) compared to C-f rats. Meanwhile, C-f rats demonstrated higher number of entries into the middle compartment (143%, *p* = 0.01) and total distance in all compartments (126%, *p* = 0.03) compared to C-m rats.

### 3.4. Synucleins mRNA Expression

**Figure 4:**
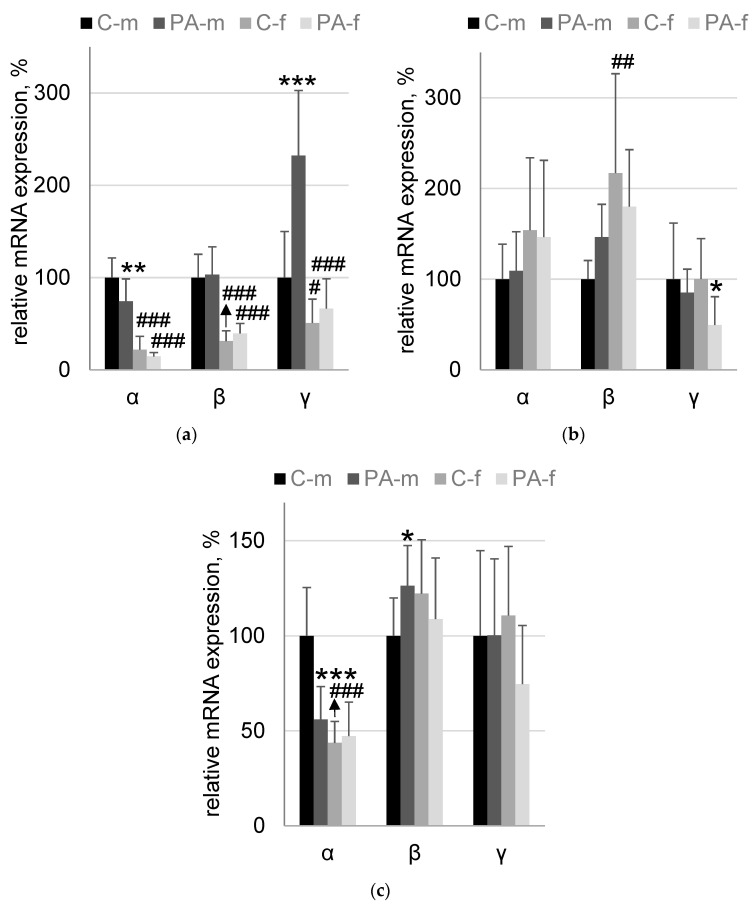
The mRNA expression levels of synucleins. (**a**)—the hippocampus; (**b**)—the hypothalamus; (**c**)—the midbrain. The corresponding members of the synuclein family are indicated by Latin letters: α, β and γ. C-m—male naïve rats, *n* = 9; PA-m—male PAE rats, *n* = 9; C-f—female naïve rats, *n* = 9; PA-f—female PAE rats, *n* = 9. Asterisk (*) indicates a significant difference between naïve and PAE rats of the same sex: *—*p* < 0.05; **—*p* < 0.01; ***—*p* < 0.001; a post hoc Duncan’s test. Hash (#) indicates a significant difference between groups of the same name of different sex: #—*p* < 0.05; ##—*p* < 0.01; ###—*p* < 0.001; post hoc Duncan’s test.

In HPC effect of PAE (F_1,32_ = 7.7; *p* = 0.009) and sex (F_1,32_ = 135; *p* = 5.29 × 10^−13^) factors on α-synuclein mRNA level was found. The expression level of *Snca* was 25.7% lower (*p* = 0.0046) in PAE-m mice compared to C-m mice. At the same time, both groups C-f and PAE-f have, respectively, 78% (*p* = 0.00006) and 60% (*p* = 0.00006) lower expression level of *Snca* compared to C-m and PAE-m mice groups, respectively. When mRNA level of β-synuclein was analyzed, only sex effect was found (F_1,32_ = 89; *p* = 9.4 × 10^−11^). Both C-f and PAE-f groups of mice had, respectively, 69% (*p* = 0.00006) and 62% (*p* = 0.00006) lower β-synuclein mRNA level compared to corresponding groups of male mice: C-m and PAE-m, respectively. The analysis of *Sncg* expression level revealed significant effects: PAE (F_1,32_ = 21; *p* = 0.00006), sex (F_1,32_ = 45; *p* = 1.4 × 10^−7^) and PAE-sex interaction (F_1,32_ = 13; *p* = 0.0009). The expression level of *Sncg* was 132% higher (*p* = 0.0001) in PAE-m mice compared to C-m mice. At the same time, both C-f and PAE-f groups of mice had, respectively, 49% (*p* = 0.046) and 71% (*p* = 0.00006) lower mRNA expression level of β-synuclein compared to corresponding groups of male mice: C-m and PA-m, respectively (Figure 4a).

In HYP effect of sex (F_1,32_ = 11.5; *p* = 0.002) on β-synuclein mRNA expression level was found. The expression level of *Sncb* was 116% higher (*p* = 0.001) in C-f mice compared to C-m group of mice. The analysis of *Sncg* expression level revealed significant effect of only PAE (F_1,32_ = 5.4; *p* = 0.03). γ-synuclein mRNA level was 51% (*p* = 0.02) lower in PAE-f group compared to C-f group of mice (Figure 4b).

In MID effect of PAE (F_1,32_ = 10.6; *p* = 0.003), sex (F_1,32_ = 27; *p* = 0.00001) and their interaction (F_1,32_ = 14.6; *p* = 0.0006) on α-synuclein mRNA content was found. The expression level of *Snca* was 44% lower (*p* = 0.0002) in PA-m mice compared to C-m mice. At the same time, the expression level of Snca was 56% lower (*p* = 0.00006) in C-f mice compared to C-m group of mice. For β-synuclein mRNA statistically significant interaction of PAE and sex effects was found (F_1,32_ = 5.3; *p* = 0.028). A tendency to increase in β-synuclein mRNA level (26%, *p* = 0.055, Duncan post-hoc test; *p* = 0.038, Fisher LSD post-hoc test) in PAE-m group compared to C-m group of mice was detected (Figure 4c).

## 4. Discussion

In humans, ethanol exposure during pregnancy is one of predictive factors of future ethanol use in the offspring [28]. Rodents exposed to alcohol prenatally are also known to exhibit high ethanol preference as well as neurodevelopmental, physiological, and behavioral deficits reviewed in [25,29]. We showed that prenatal ethanol exposure increased ethanol consumption and ethanol preference in the male but not in female offspring compared to controls. Moreover, PAE males demonstrated progressive escalation of ethanol consumption and preference of male offspring. Previous studies have shown that prenatal ethanol exposure may influence the acceptance of ethanol’s taste. The authors suggested that prenatal ethanol increases the risk to be engaged in alcohol abuse later in life because of increased preference for ethanol’s smell and taste [30]. No effect of sex of the pups on sensory responsiveness was revealed, which allowed to pool the data for both sexes [31]. Surprisingly, we found striking differences in voluntary alcohol consumption between the sexes as a response to prenatal alcohol exposure. Importantly, females of control group drank more alcohol than males. These data are in line with previously reported results of tests for alcohol intake in rats using different experimental paradigms [32]. Earlier studies suggested that sex differences in ethanol intake in rats may be due to the sex differences in brain dopamine systems believed to mediate ethanol’s reinforcing properties [33].

Here we asked whether fetal ethanol exposure can alter mRNA expression of presynaptic proteins—α-, β- and γ-synuclein known to be involved in regulation of dopamine release particularly in the mesolimbic system. We focused on three brain areas critically involved in mechanisms of alcohol-associated long-term effects—HPC, HYP and MID. Unexpectedly, PAE males had lower level of *Snca* expression in HPC and MID compared to control males, whereas no effect of PAE was found for female offspring. Interestingly, both control and PAE (to a much lesser extent) females demonstrated significantly higher levels of alcohol preference and lower levels of *Snca* expression in HIP and MID compared to males. Sex-associated difference in *Snca* expression in the brain may be explained by the effect of sex hormones including estrogen [34]. However, effect of PAE can be attributed to epigenetic alterations such as *Snca* intron 1 methylation [35]. Indeed, the inhibition of methylation in this region of *Snca* resulted in increased mRNA expression, while hypermethylation had the opposite effect [35]. One of the mechanisms involved in the regulation of *Snca* expression is dependent on the activity of DNA (cytosine-5)-methyltransferase 1 (DNMT1) [36]. This assumption is consistent with the previous results, which showed DNMT1 mRNA level is increased in the mesolimbic areas of adult PAE male rats [37]. Thus, our current data testifies that α-synuclein may be among the targets of prenatal alcohol and suggests that sex-specific long-term changes in *Snca* expression may impact the manifestation of neurobehavior disorders caused by PAE. We hypothesized several possible mechanisms behind this, which we will consider below.

It is known that α-synuclein knock-out mice have hyperdopaminergic phenotype, and knock-out of both α- and γ-synuclein genes potentiates this effect [18]. It has been estimated that the level of knockdown of endogenous α-synuclein correlates with the amount of nigral neuron loss [38]. On the contrary, decrease in α-synuclein mRNA using siRNA treatment did not cause any dramatic changes in survival of dopamine neurons or monoamine metabolism in primates [39]. These conflicting results probably reflect different levels of reduction in endogenous α-synuclein. Notably, transgenic rats overexpressing the full-length human *Snca* locus are also characterized by age-dependent degeneration of the dopaminergic system, which is preceded by the hyperdophaminergic phenotype at young age [40]. Despite early ideas about the exclusive role of the hypodopaminergic status, it is currently believed that both hypo- and hyperdopaminergia are states of vulnerability to relapse and increase context- and cue-induced alcohol-seeking behavior [41,42,43]. At the same time, literature data indicate that alcohol-dependent subjects are characterized by bidirectional changes in the content of α-synuclein [9,10]. Thus, a decrease in *Snca* expression and the predicted subsequent imbalance of the dopaminergic system may lead to a reduction in reward threshold and an increase in both alcohol preference at first presentation and the risk of relapse in the future.

A change in *Snca* expression was detected in HPC—one of the most-explored brain areas involved in complex processes such as learning and memory and emotional behavior [44]. Effects of alcohol on HPC are dependent on the developmental stage, producing pronounced alterations during gestation. It is well established that children with FASD show impaired cognition and intellectual abilities, deficient self-regulation, adaptive skills and low ability to apply the learned rules and information in the new context [45]. Hippocampal defects produced by maternal alcohol consumption during pregnancy had been well described in rodents [46,47] and can be due to a decrease in the number of neurons, an altered dendritic structure, and/or a reduced number of synapses [48,49]. In mice, an acute ethanol exposure during synaptogenesis produces apoptotic neurodegeneration in specific areas including HPC, cortex and striatum [50]. According to the findings from the animal studies α-synuclein plays an important role in the early development of synapses [51] and might act as a modulator of the size of the presynaptic vesicular pool in HPC [52]. Importantly, α-synuclein is highly expressed in the excitatory synapses marked by vesicular glutamate transporter-1 [53,54] and believed to be involved in mobilization of glutamate from the reserve pool [55]. Since glutamate plays a principle role in alcohol seeking, alcohol addiction and relapse [56,57,58] and chronic alcohol treatment promotes abnormal synaptic transmission that may lead to hippocampal cell death resulted from glutamate excitotoxicity after ethanol withdrawal [59]. Taking into account that α-synuclein knockout mice have cognitive impairments [60] we can suggest that the identified effect of downregulation of α-synuclein mRNA in HPC, perhaps, may be partly responsible for cognitive deficit which is typical for PAE offspring [61,62].

MID became another brain structure in which a change in the expression of *Snca* was detected. According to our previous data downregulation of α-synuclein mRNA expression was detected in MID of adult male rats with high initial ethanol preference [63]. It is known, a loss of α-synuclein due to the low mRNA expression or pathological aggregation may increase DA synthesis [64]. As mentioned above, this shift of the DA homeostasis to the hyperdopaminergic status may be partly responsible for hyperactivity reported in animal models of PAE [65].

We did not find any differences between PAE and control males in unconditioned anxiety-like behavior in the light-dark box. These results do not contradict the literature data, showing no significant differences in anxiety between PAE and control rats, but a pronounced increase in anxiety level in PAE males subjected to postnatal chronic mild stress [66]. At the same time, we observed decreased anxiety level in PAE females compared to control females. It should be noted that we observed this effect of PAE using the “light-dark box” test and further studies are needed to confirm the effects of PAE using a wider range of behavioral tests related to anxiety including open field and elevated plus maze. There is not much data on synucleins and anxiety behavior. It had been found that expression of α-synuclein is increased in the hippocampus HPC of rats with high levels of innate anxiety [67], however α-synuclein knock-out mice had similar to naïve mice anxiety level [68]. It looks likely that changes in α-synuclein expression are not critically involved in modulation of anxiety-related behavior. On the contrary, γ-synuclein knock-out mice characterized by decreased state anxiety and have enhancing exploratory behavior and cognitive abilities in several behavior paradigms [69], and γ-synuclein mRNA level was decreased in the HYP of PAE female rats. Thus, γ-synuclein may be responsible for alterations of emotional status of PAE female rats.

Compare to α-synuclein β- and γ-synucleins are largely understudied in the field of neuronal basis of alcohol and drug addiction. The majority of studies used overexpression or knock-out of these genes and meet difficulties in interpreting the data. It was found, that β-synuclein is upregulated in MID of mice lacking alpha-synuclein, γ-synuclein, or both α- and γ-synuclein, γ-synuclein is upregulated in mice lacking both α- and β-synuclein [70]. Even though deficiency of α-synuclein cannot be completely compensated by other members of the family [71], transcriptional activation β-synuclein in MID and γ-synuclein in HPC can be suggested as neuroadaptive mechanism. However, the opposite effect is also possible. It has been shown that increased expression of β- and γ-synuclein reduces synaptic vesicle binding of α-synuclein [72]. Relying on data that loss of α-synuclein could contribute to synaptic dysfunction in the aging brain [73], changes in the expression of β- and γ-synuclein in male PAE offspring can be interpreted as a synergistic effect leading to more pronounced synaptic dysfunction. At the same time, overexpression of γ-synuclein decreases DA neurotransmission in the nigrostriatal and mesocortical pathways, and increased γ-synuclein levels in the midbrain DA system correlated with an impaired cognitive function [74], which are typical for PAE offspring.

## 5. Conclusions

Our data show sex-specific changes in alcohol preference and mRNA expression of synucleins in rats prenatally exposed to alcohol. These facts point to the idea that alcohol consumption during gestation could alter the mechanisms of synaptic vesicle trafficking and neurotransmission in the selected brain areas, and these alterations could manifest during adulthood. Finding out how PAE changes the gene-environment interactions and determines behavioral profiles of adults including postnatal vulnerability for developing patterns of alcohol use and abuse appears to be a major goal of animal models in future research.

## Figures and Tables

**Table 1:** Oligonucleotide sequences for primers used in the qRT-PCR.

Gene	Primers	
	Forward	Reverse
β-actin	5′-cactgccg-catcctcttcct-3′	5′-aaccgctcatt-gccgatagtg-3′
*Snca* (α-synuclein)	5′-tgtcaagaaggaccagatg-3′	5′-caggctcatagtcttggtag-3′
*Sncb* (β-synuclein)	5′-agttccccacagacctgaag-3′	5′-ttacgcctctggctcgtattc-3′
*Sncg* (γ-synuclein)	5′-aaacatcgtggtcaccacc-3′	5′-tctagtctcctccactcttg-3′

## Data Availability

The data presented in this study are openly available in Mendeley Data, link Kokhan, Viktor (2022), “Sex-related differences: synuclein and prenatal alcohol exposure”, Mendeley Data, V1, doi: 10.17632/kmn44h85jg.1.

## References

[1] Gupta I., Dandavate R., Gupta P., Agrawal V., Kapoor M. (2020). Recent advances in genetic studies of alcohol use disorders. Curr. Genet. Med. Rep..

[2] Longley M.J., Lee J., Jung J., Lohoff F.W. (2021). Epigenetics of alcohol use disorder-A review of recent advances in DNA methylation profiling. Addict. Biol..

[3] Gaztanaga M., Angulo-Alcalde A., Chotro M.G. (2020). Prenatal Alcohol Exposure as a Case of Involuntary Early Onset of Alcohol Use: Consequences and Proposed Mechanisms From Animal Studies. Front. Behav. Neurosci..

[4] Jacobson J.L., Akkaya-Hocagil T., Ryan L.M., Dodge N.C., Richardson G.A., Olson H.C., Coles C.D., Day N.L., Cook R.J., Jacobson S.W. (2021). Effects of prenatal alcohol exposure on cognitive and behavioral development: Findings from a hierarchical meta-analysis of data from six prospective longitudinal U.S. cohorts. Alcohol. Clin. Exp. Res..

[5] Charness M.E. (2022). Fetal Alcohol Spectrum Disorders: Awareness to Insight in Just 50 Years. Alcohol Res..

[6] Das J. (2020). SNARE Complex-Associated Proteins and Alcohol. Alcohol. Clin. Exp. Res..

[7] Nemani V.M., Lu W., Berge V., Nakamura K., Onoa B., Lee M.K., Chaudhry F.A., Nicoll R.A., Edwards R.H. (2010). Increased expression of alpha-synuclein reduces neurotransmitter release by inhibiting synaptic vesicle reclustering after endocytosis. Neuron.

[8] Cahill C.M., Aleyadeh R., Gao J., Wang C., Rogers J.T. (2020). Alpha-Synuclein in Alcohol Use Disorder, Connections with Parkinson’s Disease and Potential Therapeutic Role of 5’ Untranslated Region-Directed Small Molecules. Biomolecules.

[9] Levey D.F., Le-Niculescu H., Frank J., Ayalew M., Jain N., Kirlin B., Learman R., Winiger E., Rodd Z., Shekhar A. (2014). Genetic risk prediction and neurobiological understanding of alcoholism. Transl. Psychiatry.

[10] Janeczek P., MacKay R.K., Lea R.A., Dodd P.R., Lewohl J.M. (2014). Reduced expression of alpha-synuclein in alcoholic brain: Influence of SNCA-Rep1 genotype. Addict. Biol..

[11] Bonsch D., Reulbach U., Bayerlein K., Hillemacher T., Kornhuber J., Bleich S. (2004). Elevated alpha synuclein mRNA levels are associated with craving in patients with alcoholism. Biol. Psychiatry.

[12] Foroud T., Wetherill L.F., Liang T., Dick D.M., Hesselbrock V., Kramer J., Nurnberger J., Schuckit M., Carr L., Porjesz B. (2007). Association of alcohol craving with alpha-synuclein (SNCA). Alcohol. Clin. Exp. Res..

[13] Rotermund C., Reolon G.K., Leixner S., Boden C., Bilbao A., Kahle P.J. (2017). Enhanced motivation to alcohol in transgenic mice expressing human alpha-synuclein. J. Neurochem..

[14] Liang T., Kimpel M.W., McClintick J.N., Skillman A.R., McCall K., Edenberg H.J., Carr L.G. (2010). Candidate genes for alcohol preference identified by expression profiling in alcohol-preferring and -nonpreferring reciprocal congenic rats. Genome Biol..

[15] Janowska M.K., Wu K.P., Baum J. (2015). Unveiling transient protein-protein interactions that modulate inhibition of alpha-synuclein aggregation by beta-synuclein, a pre-synaptic protein that co-localizes with alpha-synuclein. Sci. Rep..

[16] Buchman V.L., Hunter H.J., Pinon L.G., Thompson J., Privalova E.M., Ninkina N.N., Davies A.M. (1998). Persyn, a member of the synuclein family, has a distinct pattern of expression in the developing nervous system. J. Neurosci. Off. J. Soc. Neurosci..

[17] Kokhan V.S., Kokhan T.Y.G., Samsonova A.N., Fisenko V.P., Ustyugov A.A., Aliev G. (2018). The Dopaminergic Dysfunction and Altered Working Memory Performance of Aging Mice Lacking Gamma-synuclein Gene. CNS Neurol. Disord. Drug Targets.

[18] Senior S.L., Ninkina N., Deacon R., Bannerman D., Buchman V.L., Cragg S.J., Wade-Martins R. (2008). Increased striatal dopamine release and hyperdopaminergic-like behaviour in mice lacking both alpha-synuclein and gamma-synuclein. Eur. J. Neurosci..

[19] Wise R.A., Jordan C.J. (2021). Dopamine, behavior, and addiction. J. Biomed. Sci..

[20] Nutt D., Hayes A., Fonville L., Zafar R., Palmer E.O.C., Paterson L., Lingford-Hughes A. (2021). Alcohol and the Brain. Nutrients.

[21] White A.M. (2020). Gender Differences in the Epidemiology of Alcohol Use and Related Harms in the United States. Alcohol Res..

[22] Rossetti M.G., Patalay P., Mackey S., Allen N.B., Batalla A., Bellani M., Chye Y., Cousijn J., Goudriaan A.E., Hester R. (2021). Gender-related neuroanatomical differences in alcohol dependence: Findings from the ENIGMA Addiction Working Group. Neuroimage Clin..

[23] Flores-Bonilla A., De Oliveira B., Silva-Gotay A., Lucier K.W., Richardson H.N. (2021). Shortening time for access to alcohol drives up front-loading behavior, bringing consumption in male rats to the level of females. Biol. Sex Differ..

[24] Datta U., Schoenrock S.E., Bubier J.A., Bogue M.A., Jentsch J.D., Logan R.W., Tarantino L.M., Chesler E.J. (2020). Prospects for finding the mechanisms of sex differences in addiction with human and model organism genetic analysis. Genes Brain Behav..

[25] Patten A.R., Fontaine C.J., Christie B.R. (2014). A comparison of the different animal models of fetal alcohol spectrum disorders and their use in studying complex behaviors. Front. Pediatr..

[26] West J.R. (1987). Fetal alcohol-induced brain damage and the problem of determining temporal vulnerability: A review. Alcohol Drug Res..

[27] Quinn R. (2005). Comparing rat’s to human’s age: How old is my rat in people years?. Nutrition.

[28] Baer J.S., Sampson P.D., Barr H.M., Connor P.D., Streissguth A.P. (2003). A 21-year longitudinal analysis of the effects of prenatal alcohol exposure on young adult drinking. Arch. Gen. Psychiatry.

[29] Almeida L., Andreu-Fernandez V., Navarro-Tapia E., Aras-Lopez R., Serra-Delgado M., Martinez L., Garcia-Algar O., Gomez-Roig M.D. (2020). Murine Models for the Study of Fetal Alcohol Spectrum Disorders: An Overview. Front. Pediatr..

[30] Youngentob S.L., Glendinning J.I. (2009). Fetal ethanol exposure increases ethanol intake by making it smell and taste better. Proc. Natl. Acad. Sci. USA.

[31] Glendinning J.I., Simons Y.M., Youngentob L., Youngentob S.L. (2012). Fetal ethanol exposure attenuates aversive oral effects of TrpV1, but not TrpA1 agonists in rats. Exp. Biol. Med..

[32] Li J., Chen P., Han X., Zuo W., Mei Q., Bian E.Y., Umeugo J., Ye J. (2019). Differences between male and female rats in alcohol drinking, negative affects and neuronal activity after acute and prolonged abstinence. Int. J. Physiol. Pathophysiol. Pharmacol..

[33] Blanchard B.A., Glick S.D. (1995). Sex differences in mesolimbic dopamine responses to ethanol and relationship to ethanol intake in rats. Recent Dev. Alcohol..

[34] Spence J.P., Reiter J.L., Qiu B., Gu H., Garcia D.K., Zhang L., Graves T., Williams K.E., Bice P.J., Zou Y. (2018). Estrogen-Dependent Upregulation of Adcyap1r1 Expression in Nucleus Accumbens Is Associated With Genetic Predisposition of Sex-Specific QTL for Alcohol Consumption on Rat Chromosome 4. Front. Genet..

[35] Pavlou M.A.S., Pinho R., Paiva I., Outeiro T.F. (2017). The yin and yang of alpha-synuclein-associated epigenetics in Parkinson’s disease. Brain A J. Neurol..

[36] Desplats P., Spencer B., Coffee E., Patel P., Michael S., Patrick C., Adame A., Rockenstein E., Masliah E. (2011). Alpha-synuclein sequesters Dnmt1 from the nucleus: A novel mechanism for epigenetic alterations in Lewy body diseases. J. Biol. Chem..

[37] Razumkina E., Anokhin P., Sarycheva N., Shamakina I. (2019). Prenatal alcohol exposure increases DNA-methyltransferases 1 and 3a its mRNA levels in the rat mesolimbic brain areas. Eur. Neuropsychopharmacol..

[38] Gorbatyuk O.S., Li S., Nash K., Gorbatyuk M., Lewin A.S., Sullivan L.F., Mandel R.J., Chen W., Meyers C., Manfredsson F.P. (2010). In vivo RNAi-mediated alpha-synuclein silencing induces nigrostriatal degeneration. Mol. Ther. J. Am. Soc. Gene Ther..

[39] McCormack A.L., Mak S.K., Henderson J.M., Bumcrot D., Farrer M.J., Di Monte D.A. (2010). Alpha-synuclein suppression by targeted small interfering RNA in the primate substantia nigra. PLoS ONE.

[40] Polissidis A., Koronaiou M., Kollia V., Koronaiou E., Nakos-Bimpos M., Bogiongko M., Vrettou S., Karali K., Casadei N., Riess O. (2021). Psychosis-Like Behavior and Hyperdopaminergic Dysregulation in Human alpha-Synuclein BAC Transgenic Rats. Mov. Disord..

[41] Hirth N., Meinhardt M.W., Noori H.R., Salgado H., Torres-Ramirez O., Uhrig S., Broccoli L., Vengeliene V., Rossmanith M., Perreau-Lenz S. (2016). Convergent evidence from alcohol-dependent humans and rats for a hyperdopaminergic state in protracted abstinence. Proc. Natl. Acad. Sci. USA.

[42] Hansson A.C., Grunder G., Hirth N., Noori H.R., Spanagel R., Sommer W.H. (2019). Dopamine and opioid systems adaptation in alcoholism revisited: Convergent evidence from positron emission tomography and postmortem studies. Neurosci. Biobehav. Rev..

[43] Hauser S.R., Mulholland P.J., Truitt W.A., Waeiss R.A., Engleman E.A., Bell R.L., Rodd Z.A. (2021). Adolescent Intermittent Ethanol (AIE) Enhances the Dopaminergic Response to Ethanol within the Mesolimbic Pathway during Adulthood: Alterations in Cholinergic/Dopaminergic Genes Expression in the Nucleus Accumbens Shell. Int. J. Mol. Sci..

[44] Santangelo V., Cavallina C., Colucci P., Santori A., Macri S., McGaugh J.L., Campolongo P. (2018). Enhanced brain activity associated with memory access in highly superior autobiographical memory. Proc. Natl. Acad. Sci. USA.

[45] Wilhoit L.F., Scott D.A., Simecka B.A. (2017). Fetal Alcohol Spectrum Disorders: Characteristics, Complications, and Treatment. Community Ment Health J..

[46] An L., Zhang T. (2013). Spatial cognition and sexually dimorphic synaptic plasticity balance impairment in rats with chronic prenatal ethanol exposure. Behav. Brain Res..

[47] An L., Zhang T. (2015). Prenatal ethanol exposure impairs spatial cognition and synaptic plasticity in female rats. Alcohol.

[48] Guerri C., Bazinet A., Riley E.P. (2009). Foetal Alcohol Spectrum Disorders and alterations in brain and behaviour. Alcohol Alcohol..

[49] Bon L.I., Zimatkin S.M. (2017). Disruption of synaptogenesis in the rats brain cortex after antenatal alcoholisation. J. Grodno State Med. Univ..

[50] Sadrian B., Lopez-Guzman M., Wilson D.A., Saito M. (2014). Distinct neurobehavioral dysfunction based on the timing of developmental binge-like alcohol exposure. Neuroscience.

[51] Hsu L.J., Mallory M., Xia Y., Veinbergs I., Hashimoto M., Yoshimoto M., Thal L.J., Saitoh T., Masliah E. (1998). Expression pattern of synucleins (non-Abeta component of Alzheimer’s disease amyloid precursor protein/alpha-synuclein) during murine brain development. J. Neurochem..

[52] Murphy D.D., Rueter S.M., Trojanowski J.Q., Lee V.M. (2000). Synucleins are developmentally expressed, and alpha-synuclein regulates the size of the presynaptic vesicular pool in primary hippocampal neurons. J. Neurosci..

[53] Taguchi K., Watanabe Y., Tsujimura A., Tatebe H., Miyata S., Tokuda T., Mizuno T., Tanaka M. (2014). Differential expression of alpha-synuclein in hippocampal neurons. PLoS ONE.

[54] Taguchi K., Watanabe Y., Tsujimura A., Tanaka M. (2019). Expression of alpha-synuclein is regulated in a neuronal cell type-dependent manner. Anat. Sci. Int..

[55] Gureviciene I., Gurevicius K., Tanila H. (2007). Role of alpha-synuclein in synaptic glutamate release. Neurobiol. Dis..

[56] Cheng H., Kellar D., Lake A., Finn P., Rebec G.V., Dharmadhikari S., Dydak U., Newman S. (2018). Effects of Alcohol Cues on MRS Glutamate Levels in the Anterior Cingulate. Alcohol Alcohol..

[57] Ceccarini J., Leurquin-Sterk G., Crunelle C.L., de Laat B., Bormans G., Peuskens H., Van Laere K. (2020). Recovery of Decreased Metabotropic Glutamate Receptor 5 Availability in Abstinent Alcohol-Dependent Patients. J. Nucl. Med..

[58] Burnette E.M., Nieto S.J., Grodin E.N., Meredith L.R., Hurley B., Miotto K., Gillis A.J., Ray L.A. (2022). Novel Agents for the Pharmacological Treatment of Alcohol Use Disorder. Drugs.

[59] Gerace E., Landucci E., Bani D., Moroni F., Mannaioni G., Pellegrini-Giampietro D.E. (2018). Glutamate Receptor-Mediated Neurotoxicity in a Model of Ethanol Dependence and Withdrawal in Rat Organotypic Hippocampal Slice Cultures. Front. Neurosci..

[60] Kokhan V.S., Afanasyeva M.A., Van’kin G.I. (2012). alpha-Synuclein knockout mice have cognitive impairments. Behav. Brain Res..

[61] Mattson S.N., Crocker N., Nguyen T.T. (2011). Fetal alcohol spectrum disorders: Neuropsychological and behavioral features. Neuropsychol. Rev..

[62] Olguin S.L., Thompson S.M., Young J.W., Brigman J.L. (2021). Moderate prenatal alcohol exposure impairs cognitive control, but not attention, on a rodent touchscreen continuous performance task. Genes Brain Behav..

[63] Anokhin P.K., Shamakina I.Y., Ustyugov A.A., Bachurin S.O., Proskuryakova T.V. (2016). A comparison of the expression of α-synuclein mRNA in the brain of rats with different levels of alcohol consumption. Neurochem. J..

[64] Tehranian R., Montoya S.E., Van Laar A.D., Hastings T.G., Perez R.G. (2006). Alpha-synuclein inhibits aromatic amino acid decarboxylase activity in dopaminergic cells. J. Neurochem..

[65] Hausknecht K.A., Acheson A., Farrar A.M., Kieres A.K., Shen R.Y., Richards J.B., Sabol K.E. (2005). Prenatal alcohol exposure causes attention deficits in male rats. Behav. Neurosci..

[66] Hellemans K.G., Verma P., Yoon E., Yu W., Weinberg J. (2008). Prenatal alcohol exposure increases vulnerability to stress and anxiety-like disorders in adulthood. Ann. N. Y. Acad. Sci..

[67] Chiavegatto S., Izidio G.S., Mendes-Lana A., Aneas I., Freitas T.A., Torrao A.S., Conceicao I.M., Britto L.R., Ramos A. (2009). Expression of alpha-synuclein is increased in the hippocampus of rats with high levels of innate anxiety. Mol. Psychiatry.

[68] Pena-Oliver Y., Buchman V.L., Stephens D.N. (2010). Lack of involvement of alpha-synuclein in unconditioned anxiety in mice. Behav. Brain Res..

[69] Kokhan V.S., Van’kin G.I., Bachurin S.O., Shamakina I.Y. (2013). Differential involvement of the gamma-synuclein in cognitive abilities on the model of knockout mice. BMC Neurosci..

[70] Chandra S., Fornai F., Kwon H.B., Yazdani U., Atasoy D., Liu X., Hammer R.E., Battaglia G., German D.C., Castillo P.E. (2004). Double-knockout mice for alpha- and beta-synucleins: Effect on synaptic functions. Proc. Natl. Acad. Sci. USA.

[71] Connor-Robson N., Peters O.M., Millership S., Ninkina N., Buchman V.L. (2016). Combinational losses of synucleins reveal their differential requirements for compensating age-dependent alterations in motor behavior and dopamine metabolism. Neurobiol. Aging.

[72] Carnazza K.E., Komer L.E., Xie Y.X., Pineda A., Briano J.A., Gao V., Na Y., Ramlall T., Buchman V.L., Eliezer D. (2022). Synaptic vesicle binding of alpha-synuclein is modulated by beta- and gamma-synucleins. Cell Rep..

[73] Mak S.K., McCormack A.L., Langston J.W., Kordower J.H., Di Monte D.A. (2009). Decreased alpha-synuclein expression in the aging mouse substantia nigra. Exp. Neurol..

[74] Pavia-Collado R., Rodriguez-Aller R., Alarcon-Aris D., Miquel-Rio L., Ruiz-Bronchal E., Paz V., Campa L., Galofre M., Sgambato V., Bortolozzi A. (2022). Up and Down gamma-Synuclein Transcription in Dopamine Neurons Translates into Changes in Dopamine Neurotransmission and Behavioral Performance in Mice. Int. J. Mol. Sci..

